# Feasibility, user satisfaction, and knowledge improvement after a VR training program for healthcare professionals managing behavioral and psychological symptoms of dementia (BPSD): Protocol for the FORMSPC-REALVI single-arm pre-post study

**DOI:** 10.1371/journal.pone.0325910

**Published:** 2025-06-10

**Authors:** Hermine Lenoir, Maribel Pino, Sébastien Dacunha, Anne-Sophie Rigaud

**Affiliations:** 1 Université Paris-Cité, INSERM Optimisation thérapeutique en pharmacologie OTEN U1144, Paris, France; 2 Geriatric Department 1&2. Resources and Research Memory Center (Centre Mémoire de Ressources et de Recherches) Ile de France – Broca Hospital, Assistance Publique - Hôpitaux de Paris, F-75013, Paris, France; 3 Broca Living Lab, Hôpital Broca, Paris, France; 4 CEN STIMCO, 54 rue Pascal, Paris, France; PLOS: Public Library of Science, UNITED KINGDOM OF GREAT BRITAIN AND NORTHERN IRELAND

## Abstract

**Background:**

Behavioral and psychological symptoms are a common challenge for healthcare professionals when managing patients with dementia, and effective verbal and nonverbal communication skills are crucial in caring for such patients.

**Objectives:**

This article describes a research study protocol for investigating the effectiveness of a virtual reality (VR) training program for healthcare professionals in managing disruptive behavioral and psychological symptoms of dementia (BPSD), such as aggressiveness, agitation, and care refusal.

**Methods:**

The training scenarios were co-designed with ten healthcare professionals and implemented using an immersive 3D VR platform. Forty geriatric healthcare professionals will participate in a 2-hour training session using VR movies and a Moodle-based theoretical reinforcement. Before and after the training, participants will complete self-assessment questionnaires and knowledge-based quizzes designed to evaluate their perceived competence and understanding of appropriate communication strategies with patients displaying BPSD. The primary outcome will be the change in quiz scores between the pre- and post-training evaluations. Secondary outcomes include training satisfaction, perceived competence, and system usability.

**Hypotheses:**

**Trial registration:**

This study is registered in the French General Data Protection Regulation (GDPR) registry of Assistance Publique – Hôpitaux de Paris (N° 2022 0518135339–18 May 2022). As the trial targets health-providers and measures effects only on them (and not on providers’ patients), clinical trial registration is not required (see ICMJE guidelines: https://www.icmje.org/about-icmje/faqs/clinical-trials-registration/).

We hypothesize that the VR training program will lead to a significant improvement in participants’ knowledge and self-perceived competence in managing disruptive BPSD. This study will provide valuable insights into the feasibility and educational value of VR in geriatric communication training. If proven effective, VR training can be a valuable tool in training healthcare professionals to manage challenging behaviors in patients with dementia, ultimately leading to better quality of care for patients.

## Introduction

Neurocognitive disorders (Dementia) are debilitating conditions that affect some 55 million people worldwide, in particular in aging societies, and nearly 10 million new cases each year [1]. During the clinical course of the disease, beside cognitive components (language, memory, judgment, and reasoning impairments) [2], behavioral and psychological symptoms are common [3], and severely worsen the suffering of the patient and caregiver burden [4,5].

The disruptive behavioral and psychological symptoms of dementia (BPSD) such as agitation, aggressiveness, opposition, and care refusal, are often subtended by environmental factors, sleep deprivation [6], or by *delirium* (acute confusional state) and favored by anxiety, depression, delusions and/or hallucinations. Psychotropic drugs to treat such disorders, in this particularly frail population, are often source of iatrogenic effects (drowsiness, falls, faecal impaction, acute urine retention, vascular accidents, extrapyramidal symptoms, etc…) [7], or sometimes ineffective [8,9]. Therefore, the use of a first line nonpharmacologic approach is highly recommended by health authorities and guidelines.

Professional healthcaregivers of people living with dementia are often faced with challenging situations (such as opposition/care refusal, aggressiveness, agitation…) where their verbal and nonverbal skills can make all the difference in the quality of care that they provide. Insufficient skills of professionals to manage difficult situations, increase the risk of negative interactions, exhaustion, absenteeism, inappropriate practices (*i.e*., physical restraint) [10], and even abuse [11,12]. They are also responsible for resort to emergency or crisis services [13], increase in the average length of stay at hospital and consequently in healthcare costs [14,15].

Know-how to do and know-how to be (by appropriate prosody, body language, eye contact, and facial expressions) with such patients, based on validation therapy, empathetic communication, active listening, relational touch, very often allow to resolve complex situations while ensuring a better quality of life for the care recipients as well as a greater sense of competence and lower risk of burn out in professional caregivers [16]. Factors such as eye contact, physical proximity, and the clinician’s posture leaning towards the patient have been suggested to influence the duration of patient-caregiver interactions and length of the visit, patient perceived empathy, and feeling of trust.

Understanding the role of these nonverbal behaviors in patient-caregiver interactions could help optimize communication and improve patient outcomes. [17–19].

However, training for these skills can be difficult to implement in real-life scenarios. Indeed, observing the trainer interact with an agitated or aggressive patient can cause anxiety reactions in the learner. A challenging interaction for a healthcare professional already overstretched by the healthcare system may also increase levels of stress, anxiety, and anger, which in turn will affect teaching performance and communication. Moreover, the different clinical situations do not always arise when the learner and the healthcare professional are both available. Institutional contingencies, in particular reduced budget allocation for lifelong learning, but also the lack of didactic material and scarce human resources in medical and nursing services or geriatric hospitals, are some of other barriers for caregiver training [20].

The COVID-19 pandemic context, before or without the availability of effective vaccination strategies, has increased these difficulties by mandatory barrier gestures and precautionary measures’ respect, including physical distancing, limiting the clinical exposure and expert supervision required for communication skills’ acquisition, essential for an adequate care. In addition, theoretical training can be a source of weariness for learners and is generally less favored than training in real situations by caregivers in their practice over time. Interactive training, such as roleplaying, can improve these aspects but is subject to the constraints indicated in the previous paragraph.

Recent advances in educational technologies offer a growing number of innovative learning opportunities thanks to new tools. Among these, virtual reality represents a promising area with great potential for improving the training of health professionals [21,22].

VR training provides a rich, interactive, and engaging educational context, thus promoting experiential learning. It contributes to the interest and motivation of learners and effectively supports the acquisition and transfer of skills, since the learning process can be regulated in an experiential setting [22]. Current applications of virtual training in healthcare are diverse depending on their technological/multimedia sophistication, types of skills being trained (telesurgical applications, interactive simulations of the human body or brain, virtual worlds for emergency training) [23,24]. Other interesting applications include the development of immersive 3D environments for training psychiatrists and psychologists in the treatment of mental disorders [25]. The scientific literature on the contribution of VR to learning techniques (clinical reasoning and self-assessment) is increasingly rich [26].

Recently, virtual reality has been used to develop simulation and training tools, in particular through embodied virtual patients (VP) offering the opportunity to engage a safe and controlled setting for healthcare professionals to have face-to-face human-like interactions with virtual patients [17]. However, the development of simulation tools for training clinicians to communicate with people with dementia is still very limited [27]. A study conducted by Stargatt *et al.* found that healthcare professionals who received VR training had higher levels of empathy towards patients with dementia compared to those who received traditional training [28]. Empathy is a critical component of effective communication with people with dementia, as it allows healthcare professionals to understand and meet the needs of the patient in a compassionate and effective manner.

Orton *et al.* from the University of Iowa’s showed that a web-based platform called *GeriaSims* virtual patient program offered clinicians the opportunity of interacting with a virtual patient embodied as an elderly person and was effective for geriatric education. Indeed, more than 85% of the responses to an evaluation survey of *GeriaSims* users indicated favorable perceptions of instructional effectiveness, efficiency, and ease of use [29]. In another study, Robinson *et al*. focused on training communication skills of 82 speech pathology students with a virtual elderly resident of a nursing home with behavioral symptoms of dementia [30]. The two successive 15-minute interactions were based on predetermined scenarios of verbal (e.g., comprehension difficulties, word search, confusion) and nonverbal (e.g., crying, shrugging, chuckling) responses of the virtual elderly that were representative of dementia. The analysis of the trainee’s verbal and nonverbal (V/NV) behavior coupled with a self-rating by the trainees of their communication skills revealed an improvement in students’ communication skills in the second interaction. However, it was not possible to distinguish the benefit of the simulation on the verbal versus nonverbal level.

The few studies on VR with virtual patients in psychiatric or geriatric settings, can be divided in two categories with different results [17]: 1- Tools based on web interfaces or pre-recorded videos (associated with text-based interfaces) which do not allow for realistic humanlike conversations and may lack realism and fluidity; 2- Immersive experiences using virtual patients displayed on human-sized screens and interacting in natural language [30–34], which are rarely fully autonomous and currently require the intervention of a human WoZ operator [35–40].

Amongst these studies, a few focused on the evaluation of the learner and the assessments performed included essentially feasibility and usability of simulation tool itself. Chaby *et al*. [17], in their overview paper on embodied virtual patient as a simulation-based framework for training clinician-patient communication skills in geriatric and psychiatric settings, noticed that an aspect seldom assessed in studies was a finegrade analysis of the learner’s verbal and nonverbal behaviors which play a crucial role in clinician-patient relationships [41]. The authors suggest that the link between such precise analysis of verbal and nonverbal interactions and learning gain of trainees to be investigated [17].

While the use of virtual reality for training in dementia care is expanding, most existing studies have focused on feasibility and user satisfaction rather than on pedagogical effectiveness or skill acquisition. Recent research supports the potential of immersive VR training to improve not only empathy but also self-efficacy among healthcare professionals in emotionally complex caregiving scenarios, including dementia-related aggression and care refusal [42,43]. However, limitations such as the lack of interaction with avatars and the absence of long-term impact assessments persist. As highlighted in recent reviews, successful implementation of VR programs in geriatric settings also requires attention to technical support, user acceptability, and integration within the broader care environment [44]. These elements are essential to optimize both the educational and organizational value of such programs.

In the following presented protocol, the aim of our study is to assess the pedagogical effectiveness, usability, and user satisfaction of a virtual reality (VR) training program designed to improve verbal and nonverbal communication skills in geriatric healthcare professionals managing patients with BPSD. The study uses a single-arm, pre-post design to measure individual progression in knowledge and perceived competence following the intervention.

Given the exploratory nature of this pedagogical intervention and the limited availability of healthcare staff, a single-arm, pre-post design was chosen. Although this design does not allow for a direct comparison with traditional training, it enables a rigorous evaluation of individual knowledge progression and perceived competence following the VR-based program.

## Materials and methods

### Aims

This article details different steps of design and assessment of a VR 3D training program to promote appropriate verbal and nonverbal interaction skills of healthcare professionals in geriatric settings, specifically in managing patients with disrupted BPSD. For this purpose, we relied on the review by Verschueren *et al.*: “Developing Theory-Driven, Evidence-Based Serious Games for Health: Framework Based on Research Community Insights” [45]. The authors conducted a literature review and analyzed the insights of experts from the research community to identify the key factors that contribute to the success of serious games in promoting health. They found that theory-driven serious games, which are grounded in health behavior change theories and models, and those based on scientific evidence, are more effective in promoting health behavior change than traditional ones [45].

Based on their findings, Verschueren et al. proposed a framework that consists of four main phases: (1) conceptualization and design, (2) development and testing, (3) evaluation and optimization, and (4) dissemination and implementation. The framework emphasizes the importance of involving stakeholders, including game developers, researchers, healthcare professionals, and end-users, throughout the development process [45]. Following the guidance of these authors, we have developed a plan for designing a training program for nurses and certified nursing assistants in verbal and nonverbal communication skills with patients suffering from disruptive psychobehavioral symptoms.

Our program features a virtual professional caregiver interacting with a patient played by an actor, with various realistic scenarios, reflecting pedagogically appropriate and inappropriate verbal and nonverbal communication. The aim is to provide more effective communication training than traditional methods. The scenarios simulate situations in which patients exhibit agitation, aggressiveness, or care refusal. At the end of each module, the program offers immediate feedback on the effectiveness of the user’s communication approach, enabling them to adapt their approach to the situation. This feedback mechanism is a key advantage of VR training, providing personalized feedback that is often lacking in real-life training scenarios.

We have gathered the necessary questionnaires and evaluations to assess the feasibility and, usability of the program. The effectiveness of the program will also be assessed by measuring trainees’ satisfaction, their self-perceived competence, and the pedagogical value of the VR training program (knowledge acquisition). Our theory is that the program will empower healthcaregivers to develop non-pharmacological management skills for BPSD independently, without being constrained by space or time, in a secure environment where they will be not judged by their colleagues or superiors, and at a lower cost. This training will also provide high-quality instruction that can be customized for caregivers in a range of care settings, such as nursing homes, hospitals, and residences.

### Design and setting

This study adopts a single-arm, pre-post design to evaluate the usability, user satisfaction, self-perceived competence, and knowledge acquisition related to a virtual reality (VR) training program aimed at enhancing verbal and nonverbal communication skills among healthcare professionals working in a Parisian academic geriatric hospital.

All participants will undergo the same immersive VR-based training intervention. The impact of the training will be assessed by comparing each participant’s outcomes before and after the intervention, focusing specifically on self-perceived competence and knowledge acquisition related to the management of disruptive behavioral and psychological symptoms of dementia (BPSD). This design was selected due to feasibility constraints, including limited staff availability and the exploratory, educational nature of the intervention. Although no comparator group is included, the within-subject approach allows each participant to serve as their own control, thus enabling a rigorous and individualized assessment of training impact. This exploratory study is expected to provide valuable insights into the practical feasibility and pedagogical value of immersive VR training in geriatric care settings.

### Participants, sampling plan, inclusion, and exclusion criteria

**Fig 1:**
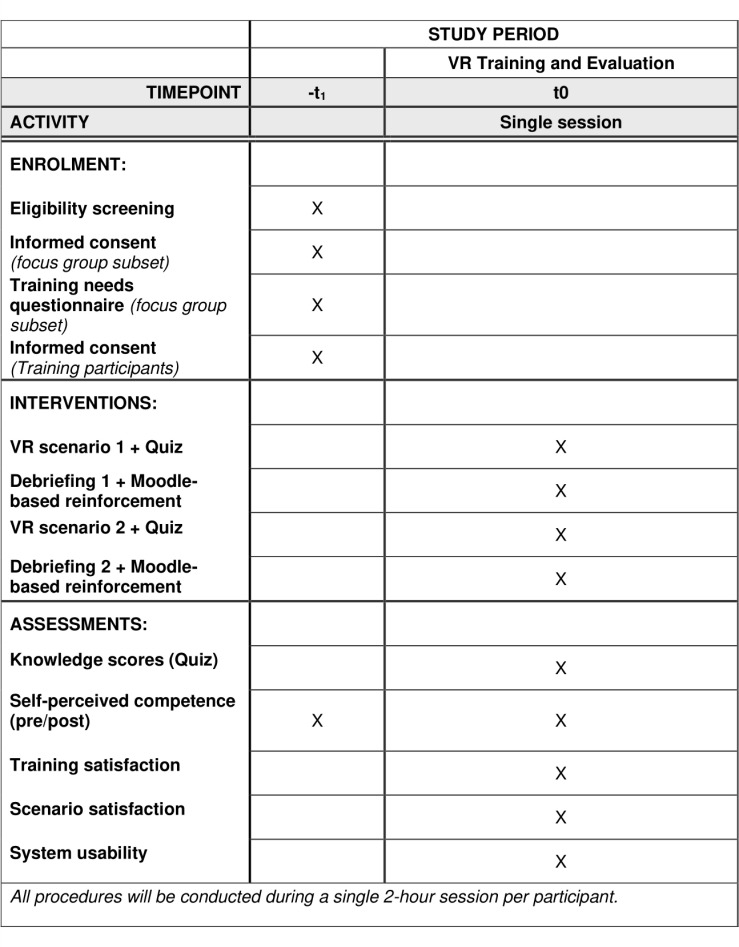
SPIRIT schedule of enrolment, intervention, and assessments.

The study will involve a total of 50 geriatric healthcare professionals, including nurses and certified nursing assistants, all working at Broca Academic Geriatric Hospital in Paris. Among them, 10 participants will be involved in a preliminary focus group dedicated to the co-design of the virtual reality training scenarios. The remaining 40 participants will take part in the main study, which follows a single-arm, pre-post design (Fig 1).

This sample size exceeds the minimum required number of 34 participants, as determined by an a priori power analysis (for a moderate effect size, dz = 0.5, with 80% power at a 5% significance level), and was increased slightly to account for potential dropouts or missing data.

Participants will be recruited on a voluntary basis via posters in the geriatric wards or during staff meetings. There will be no restrictions regarding age or gender. Healthcare professionals with a history of epilepsy, pregnant individuals, or those prone to motion sickness, balance problems, or migraines will be excluded. Both day and night shift staff are eligible.

Participants may discontinue the training session at any time, particularly in the event of discomfort, cybersickness, or any adverse reaction to the immersive VR environment. No modification of the training content is planned during the session. In case of early withdrawal, participants’ data will be excluded from the final analysis.

Data will be anonymized and will not impact any professional evaluations. No financial compensation will be provided. Psychologist evaluators will have no prior professional relationship with the participants. Each of the 40 participants in the main study will complete pre- and post-training assessments surrounding a 2-hour VR training session

**Fig 2:**
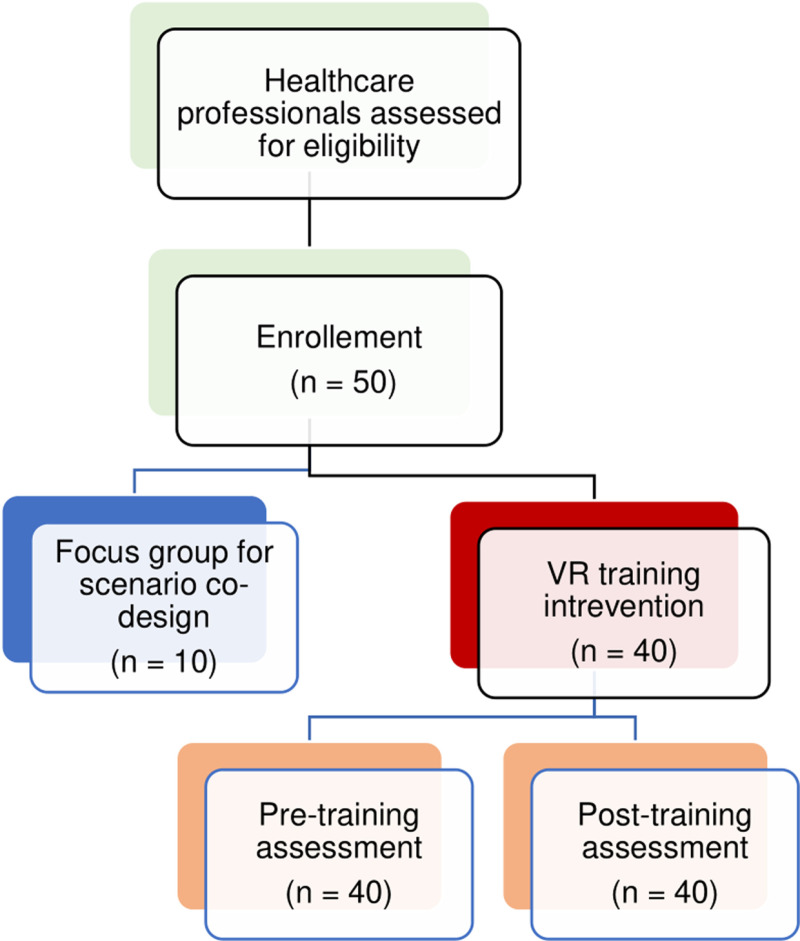
Flow chart of the study sample. Flow diagram illustrating the recruitment, inclusion, and progression of healthcare professionals throughout the VR training study.

We will ensure participants are available for focus groups, training, and evaluations. Total participation time will not exceed 120 minutes, including approximately 60 minutes for immersive virtual reality scenario viewing, quizzes, debriefing, and Moodle-based reinforcement, and 60 minutes for the completion of pre- and post-training questionnaires and evaluation forms (Fig 2). sample).

### Ethical considerations

This study was approved by the Ethics Committee for Research of Université Paris Cité (*Comité d’Éthique pour la Recherche*, IRB 00012022−25) on 17 May 2022. The study complies with all applicable national and institutional regulations.

Participants are healthcare professionals enrolled on a voluntary basis. Written informed consent will be obtained prior to any data collection or exposure to the virtual reality training program. Consent will be obtained by trained members of the research team (psychologists or medical investigators) using IRB-approved materials, including an information letter and consent form. Participants will be given sufficient time to ask questions before signing.

This study does not involve patients or animal subjects. Given its non-interventional and low-risk educational nature, no ancillary or post-trial care is planned.

### Protocol workflow

#### Step one: scenario design phase.

In this phase, 10 healthcare professionals (out of 50 participants) who have given written consent to participate in the study, will be invited to a focus group. The researchers (a couple of 2 psychologists and 2 geriatricians) will provide them two scenarios, each describing a clinical context involving a patient with disruptive BPSD (aggression, opposition, agitation, and/or anxiety) and a professional caregiver (example of a scenario in S2 Text). The scenarios will be written in a precise but concise manner, outlining situations where conflicts are likely to arise due to the patient’s non-cooperative behavior towards the caregiver (*e.g*., grooming with an unwilling patient or agitation during group activities).

Participants will be asked to provide feedback on the scenarios based on their own professional experience and knowledge, as well as to suggest modifications or additions to the dialogues and gestures of the fictional characters in order to either aggravate the conflict or soothe the patient, depending on the situation. To guarantee the pedagogical excellence of the scenarios, we will ensure that the experiences and knowledge of investigators, including doctors and psychologists, as well as healthcare personal involved in the focus group, are incorporated. Moreover, recommendations from health authorities and healthcare societies regarding the care of patients experiencing behavioral and psychological symptoms of dementia (BPSD) will also be taken into consideration. By doing so, the scenarios can be co-written with a well-rounded and comprehensive approach, taking into account various perspectives and expertise, thus ensuring their accuracy and effectiveness.

To evaluate later the pedagogical value of the scenarios, participants will be asked to propose multiple choice questions. These quizzes will be used to evaluate the subsequent participants for their ability to identify appropriate and inappropriate verbal and nonverbal communication by the fictional healthcare professional, as well as their knowledge about optimal ways to behave with demented patients with disruptive BPSD, as represented by the fictional patient in the scenarios.

Furthermore, the researchers and healthcare professionals who participate in the focus groups will collaborate in co-creating a Moodle platform. This platform will highlight the appropriate and inappropriate behaviors, potentially, employed when communicating with patients based on the scenarios.

#### Step two: VR movie design.

Based on the two scenarios codesigned by the investigators and the 10 focus group participants, a virtual reality movie will be performed. A virtual reality software is being developed as part of the project (by engineers working in the research lab of Broca Hospital), to enable the display of virtual agents, resembling a virtual professional caregiver and a patient played by an actor, on a virtual reality helmet screen. During the viewing, the participant will be fully immersed in a 3D environment that is predetermined by the scenarios designed upon the completion of the focus group (as detailed in the Step One of the project), which could be a patient’s room, unit corridors, or a care station.

#### Step three: virtual reality training and evaluation.

Following informed consent, the 40 healthcare professionals not involved in the scenario co-design focus group will participate in a single-arm, pre-post study evaluating the effectiveness of a VR-based communication training program.

Each participant will attend one individual single-session lasting approximately 2 hours, scheduled within a 10-day period. Two sessions will be held each day (9:30–11:30 am and 2:00–4:00 pm), ensuring participant availability across day and night shifts.

At the start of the session, participants will complete the “Verbal and Nonverbal Communication Training Needs Questionnaire” (see S3 Text) to assess their self-perceived communication challenges related to the management of patients with behavioral and psychological symptoms of dementia (BPSD).

They will then proceed to view the first immersive VR scenario using a 3D headset, based on content co-developed during the scenario co-design phase. Immediately afterward, they will complete a multiple-choice quiz evaluating their understanding of verbal and nonverbal communication strategies illustrated in the scenario.

A debriefing session with psychologist investigators will follow, to discuss the participant’s quiz responses and provide tailored feedback.

Participants will then access a reinforcement module on Moodle, designed to consolidate the key theoretical concepts presented in the first scenario.

This sequence (VR viewing → quiz → debriefing → Moodle training) will be repeated for a second VR scenario, followed again by a debriefing and Moodle reinforcement.

At the end of the training session, participants will complete the post-training assessments, including:

- satisfaction with the training experience,
- perceived competence in managing BPSD,
- and perceived pedagogical effectiveness of the VR scenarios.

**Fig 3:**
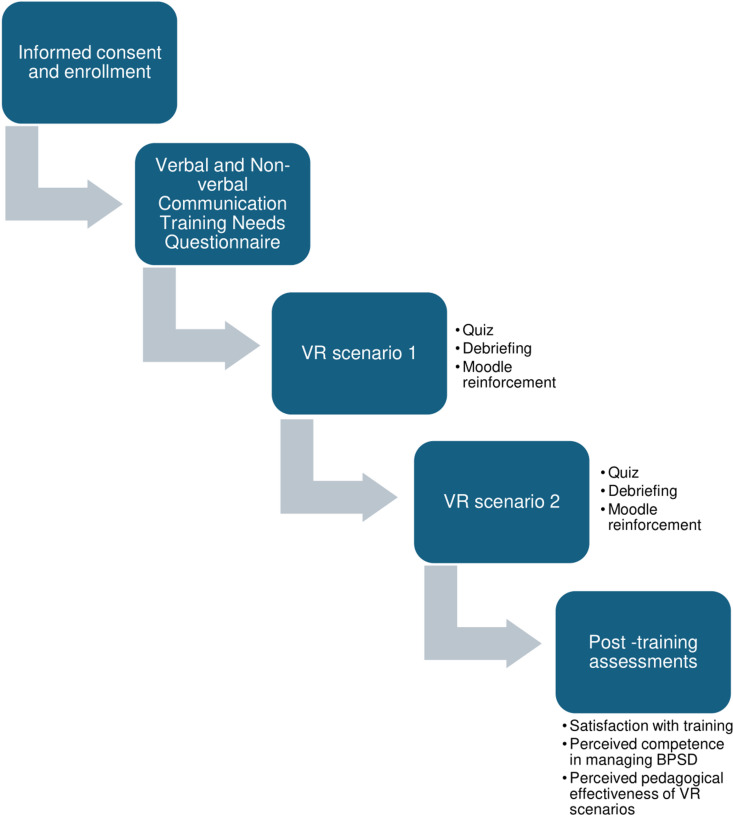
Workflow chart of the study protocol design. Overview of the sequence of procedures, including baseline questionnaires, VR scenarios, debriefings, Moodle-based reinforcement, and post-training assessments.

The full process is presented in Fig 3.

#### Step four: assessments.

After each VR scenario, participants will complete a multiple-choice quiz to assess their knowledge of appropriate verbal and nonverbal communication strategies with patients suffering from BPSD. The quiz scores, based on the percentage of correct responses, will be calculated separately for Scenario 1 and Scenario 2. These scores will be returned to participants to help track their progress and knowledge acquisition.

The primary outcome of the study, the pedagogical effectiveness of the training, will be measured by the improvement in participants’ knowledge scores between Scenario 1 and Scenario 2 (see calculation method in the *Data collection and assessment tools* section).

Participants will also complete questionnaires evaluating:

- their overall satisfaction with the training method (VR scenario movies and Moodle reinforcement),
- their self-perceived level of competence.

In addition, they will complete two supplementary questionnaires:

- one assessing satisfaction with the VR scenarios, and
- another evaluating the usability of the 3D VR training method (see details in *Data collection and assessment tools*).

The total time required to complete all assessments will not exceed 1 hour.

### Training tools

#### VR-based training program and Moodle reinforcement.

The training program combines immersive 3D virtual reality (VR) scenarios with structured Moodle-based theoretical reinforcement. The aim is to enhance participants’ ability to communicate effectively with patients displaying disruptive behavioral and psychological symptoms of dementia (BPSD), such as agitation, aggressiveness, or care refusal.

The two VR scenarios, co-designed with healthcare professionals, depict realistic care situations where verbal and nonverbal communication is challenged. After viewing each scenario, participants complete a quiz to assess their understanding of appropriate and inappropriate caregiver behaviors.

To consolidate the learning, each VR scenario is followed by a Moodle-based module. These modules provide structured theoretical content that helps participants:

- analyze the clinical situations observed in the VR scenes,
- interpret patient-caregiver interactions in light of BPSD-related challenges,
- and identify appropriate verbal and nonverbal communication strategies.

Topics covered include:

- the use of simple language, open-ended questions, and validation therapy,
- how to respond to patients with language or cognitive impairments,
- and how to manage communication during episodes of aggression or care refusal using active listening, reassurance, and de-escalation techniques.

The Moodle platform also offers guidance on behaviors to avoid, such as ambiguous gestures or rushed interactions, which can exacerbate patients’ distress. Overall, this combined approach aims to equip healthcare professionals with the knowledge, strategies, and reflective tools needed to improve their interactions with patients experiencing disruptive BPSD.

### Data collection and assessment tools

#### Evaluation of healthcare professionals’ training needs in verbal and nonverbal communication (V/NV).

At baseline, all participants will complete a self-administered training needs questionnaire specifically designed for this study (see S3 Text). The purpose of this tool is to assess the participants’ initial knowledge, training background, and expectations regarding communication with patients presenting behavioral and psychological symptoms of dementia (BPSD).

The results of this baseline assessment will help contextualize the learning process and, if necessary, fine-tune the Moodle content used to reinforce the VR scenarios.

The questionnaire includes items that evaluate the participants’ general understanding of communication challenges specific to patients with BPSD, the types of training previously completed in the field of relational care and behavioral disorders, and prior experience with digital learning technologies, including virtual reality simulation, in accordance with the recommendations of the French Haute Autorité de Santé (December 2019) [46].

Demographic information will also be collected, including age, professional role, and years of experience. This data will provide insight into the training needs across different staff profiles and contribute to the interpretation of post-training outcomes.

#### The satisfaction of participants with the training methods.

Participants’ satisfaction with the training program will be assessed at the end of the training session, following the completion of the VR scenarios, quizzes, debriefings, and Moodle modules. They will be asked to rate their overall satisfaction with the training they received using a 5-point Likert scale, where 1 indicates “very unsatisfied” and 5 indicates “very satisfied.”

This evaluation will provide insight into the perceived relevance, clarity, and usefulness of the immersive VR experience combined with theoretical reinforcement through the Moodle platform.

#### The evaluation of the participant’s satisfaction of the two VR scenarios.

All participants will be asked to complete a scenario-specific satisfaction questionnaire, developed by the research team (see S4 Text), to assess their experience with each of the two immersive VR scenarios.

This questionnaire will gather feedback on elements such as:

- the realism and relevance of the scenarios,
- the emotional engagement generated by the immersive content,
- and the perceived usefulness of the scenarios in improving real-life communication with patients with BPSD.

**To assess the usability of the VR 3D training method**, the System Usability Scale (SUS) will be employed [47]. The SUS provides a subjective viewpoint of the user on the system and its ease of use on a 5-point Likert-type scale ranging from 1 to 5. The total is calculated using the SUS questionnaire and ranges from 0 to 100, with a higher score indicating greater usability satisfaction [47].

**The learners’ self-perceived level of competence** will be quantitatively analyzed using a questionnaire created by the researchers (see S5 Text) which is based on the “perceived competence” section of the “Intrinsic Motivation Inventory” [48]. This subsection evaluates motivation and competence in a multidimensional way. The results will be reported on a 5-point Likert-type scale, ranging from 1 to 5. A higher total score indicates a greater sense of acquired competence.

**The evaluation of the pedagogical effectiveness of the training will be based on** the participants’ The pedagogical effectiveness of the training will be assessed by measuring participants’ knowledge progression between Scenario 1 and Scenario 2 of the immersive VR training. After viewing each scenario, participants will complete a multiple-choice quiz, and the learning platform will automatically calculate their scores:

- Score 1 after Scenario 1,
- Score 2 after Scenario 2.

The difference between Score 2 and Score 1 will be used as an indicator of knowledge acquisition:

- If Score 2 ≤ Score 1, no learning progress is observed,
- If Score 2 > Score 1, the participant is considered to have improved in knowledge and understanding of appropriate communication strategies.

This within-subject comparison provides a direct measure of the training tool’s educational value and its effectiveness in reinforcing verbal and nonverbal communication skills relevant to the management of patients with disruptive BPSD.

### Statistical analyses plan

To ensure the integrity and internal validity of the study, participants will be explicitly instructed not to participate in any other communication-related training during the study period. Additionally, participants will be asked to refrain from discussing the VR training content with colleagues who may also be involved in the study. Clear communication regarding these expectations will help minimize potential sources of bias or contamination.

### Primary analysis

The main objective of the analysis is to evaluate the pedagogical effectiveness of the VR training program. This will be assessed through the comparison of knowledge quiz scores completed after each scenario (Score 1 and Score 2). The mean difference between these two scores will be calculated for each participant. A paired-sample t-test will be used to determine whether the change in knowledge is statistically significant, with a significance level set at *p* < 0.05.

This test is appropriate for the within-subjects design, where each participant serves as their own control.

### Secondary analyses

Additional outcomes will be explored using both descriptive and inferential statistical methods.

- Participant satisfaction with the training program will be summarized using means and standard deviations, and further examined through frequency distributions of Likert scale responses.
- Changes in self-perceived competence before and after training will be analyzed using a paired-sample *t-*test. If normality assumptions are not met, a non-parametric alternative such as the Wilcoxon signed-rank test will be applied.
- Descriptive analyses will also be performed for system usability and scenario-specific satisfaction. Potential associations between these outcomes and participant characteristics (e.g., age, professional role, years of experience) will be assessed using correlation analyses or regression models, where appropriate.
- Additionally, exploratory subgroup analyses may be conducted to identify whether training outcomes vary according to specific participant factors, including professional role or prior exposure to virtual reality training. Predictive modeling using regression techniques may be applied if supported by the data.

### Handling missing data

Analyses will be conducted on a per-protocol basis. Participants who do not complete the post-training assessments (e.g., knowledge quizzes or self-perception questionnaires) will be excluded from the final analysis, and no imputation will be applied for missing data. Data completeness will be monitored throughout the study, and sensitivity analyses may be conducted if the extent of missing data is deemed substantial. Given the one-time, on-site nature of the intervention, participant dropout is expected to be minimal. Attendance will be recorded at the start of the session. Should a participant begin the session but fail to complete the post-training assessments, no additional data will be collected and their dataset will not be included in the analysis. To promote retention and minimize incomplete data, efforts will be made to ensure participant understanding and comfort prior to the start of the training.

### Sample size justification

The sample size was determined based on an a priori power analysis for a paired-sample design. To detect a moderate effect size (Cohen’s dz = 0.5) with 80% power at a 5% significance level using a two-tailed paired t-test, a minimum of 34 participants was required. To allow for potential dropouts or incomplete data, we increased the sample size to 40 participants. The calculation was performed using G*Power (version 3.1). We plan to include 40 participants, allowing for possible attrition.

### Software and data access

Statistical analyses will be performed using XLSTAT and G*Power (version 3.1) for power calculations. All data will be made available in accordance with the *PLOS* Data Policy upon study completion. The principal investigator and co-investigators will have full access to the final trial dataset. No contractual agreements exist that restrict the investigators’ access to the data or their ability to publish the results. In line with *PLOS One* data sharing policies, the complete study protocol, anonymized dataset, and statistical analysis files (e.g., data tables and formulas) will be made publicly available via an institutional open-access repository at the time of publication.

### Safety considerations

To ensure the safety of participants in this study, potential risks have been identified and addressed. Exposure to virtual reality, as indicated by the 2019 French National Health Security Agency (ANSES) survey, may disrupt the sensory system and cause symptoms such as nausea, dizziness, sweating, paleness, loss of balance, collectively known as “*cyber kinetosis*” [49]. These symptoms may manifest in sensitive individuals within minutes of use, and may also temporarily affect sensory, motor, and perceptive abilities, altering manual dexterity or body orientation. They also include the feeling of motion sickness or of nausea and dizziness caused by virtual reality experiences or other types of immersive technologies. Additionally, exposure to flashing lights emitted by LED screens can trigger epileptic seizures in individuals with a predisposition to them [49].

To minimize the risk of these effects, ANSES’ recommendations will be followed during this study:

- Testing with the virtual reality device will be immediately halted if participants experience symptoms such as nausea, dizziness, sweating, or paleness.
- Each participant will observe a rest period of 15 minutes during and after use of the virtual reality device.
- Participants with epilepsy or who are sensitive to motion sickness, balance issues, migraines, or pregnant women will not be included in the study (as stated in the information letter and exclusion criteria).

All adverse events related to the virtual reality training, whether solicited or spontaneously reported, will be documented by the research team using a standardized reporting form. This includes any symptoms of cybersickness (e.g., dizziness, nausea), emotional discomfort, or unexpected stress reactions during or after the sessions. The principal investigator will be responsible for reviewing and assessing all reported events in terms of severity, duration, and possible relationship to the intervention. Any serious or unexpected adverse events will be promptly reported to the institutional ethics committee in accordance with AP-HP procedures. Participants will be explicitly informed that they can report any discomfort at any time and may discontinue the session without consequence.

In the unlikely event of harm related to participation, appropriate support will be provided in accordance with institutional policies.

To minimize risks related to COVID-19, all equipment (visor, furniture) will be disinfected before and after use by each participant, and a physical distance of 2 meters will be maintained between evaluators and participants. All other barrier measures will also be strictly observed (wearing masks, using hydro-alcoholic hand sanitizer, using pens dedicated to each participant).

### Data handling

The investigator in charge of the study and data quality control will take all necessary precautions to ensure the confidentiality of information related to the research, the individuals participating in it, and the results obtained. Those involved in the research, including the investigator, are subject to professional secrecy (as defined by articles 226−13 and 226−14 of the penal code).

A randomly generated ID code will be assigned to each participant, indicating the order of subject inclusion, in accordance with regulations. The ID code will be used on documents such as assessment files to maintain confidentiality and minimise the use of personal data. The study investigator will ensure that each individual participating in the research has given written consent to access their individual data, strictly necessary for research quality control. The provisions of the General Data Protection Regulation (GDPR) will be respected, and the data collected will be stored securely on site with limited access.

In addition to the confidentiality measures already described, a structured data management plan will be implemented to ensure data quality and integrity. Data entry will be performed manually by trained research personnel using a standardized digital form. All primary outcome variables and questionnaire responses will undergo double data entry and cross-validation to minimize transcription errors. Coded datasets will be stored in encrypted Excel files with restricted access, and a version-controlled log will be maintained. Consistency and range checks will be applied at regular intervals. Only authorized team members will have access to the data via secure institutional servers at AP-HP. All data management procedures will comply with the General Data Protection Regulation (GDPR) and follow institutional data governance protocols.

### Composition, roles, responsibilities, and auditing (See S6 Text)

Given the minimal-risk, single-arm, short-term design of this educational intervention, no steering committee or endpoint adjudication committee was established. The research team itself, composed of investigators in geriatric care and psychology, was responsible for the study design, implementation, and monitoring.

No external or independent auditing of trial conduct is planned given the low-risk, single-center nature of the study. However, institutional oversight may be exercised if deemed necessary by the ethics committee.

### Status and timeline

The current study is a single-center, single-arm pre-post protocol evaluating a virtual reality (VR) training program for healthcare professionals in a geriatric hospital in Paris (Broca Hospital, Assistance Publique – Hôpitaux de Paris). Ethical approval has been obtained, and the study protocol has been finalized.

Participant recruitment has not yet started; it is scheduled to begin in June 2025 and to be completed by July 2025. Data collection will take place from September to December 2025, and preliminary results are expected by January 2026.

A preliminary set of training scenarios has been drafted and will be refined through a focus group involving participating professionals at the beginning of the study.

All data generated or analyzed during this study will be made available upon reasonable request, with access details included in the final publication.

## Discussion

### Strenghts of study protocol design

We believe that our study protocol is particularly interesting and original when compared to other training programs. Unlike traditional approaches, our method does not require direct interaction between the trainee and the patient. Instead, the trainee is tasked with observing and analyzing the attitudes and verbal interactions of a virtual caregiver colleague as they interact with the fictional patient. By learning from the mistakes made by their peers during these virtual interactions, trainees can gain valuable experience and be better equipped to handle similar situations in the future.

In addition, we maintain that our decision to use a real actor, rather than an avatar, adds another layer of authenticity to the training process. We believe that this approach will enable trainee caregivers to gain a deeper appreciation of the reality of their daily work.

Previous research has shown that virtual reality training can effectively enhance communication abilities among healthcare professionals who work with patients affected by dementia and other cognitive impairments [50,51]. Furthermore, a study by Lee et al. found that simulation-based training can enhance care providers’ empathy towards dementia patients [52]. Our study builds on these findings by utilizing virtual reality training to improve communication skills in a unique and innovative way.

By incorporating effective communication and practical skills, our program aims to provide healthcare professionals with exposure to potentially stressful situations that can facilitate the development of emotional and stress management techniques, mitigating the risk of burnout while also promoting optimal care practices. The program modules are designed to be utilized in self-paced, independent e-learning sessions, or alternatively in supervised group sessions if required by a psychologist or other expert trainers.

We believe that our protocol will be of significant interest as it addresses a current limitation in the development of simulation tools for training clinicians to communicate with people with dementia. While previous studies have primarily focused on assessing feasibility and user satisfaction [51], here remains a critical need for rigorous evaluations of the pedagogical effectiveness of VR training programs using structured and transparent methodologies. Although our study does not adopt a randomized controlled design, it offers a robust within-subject pre-post approach, which is particularly well suited to exploring knowledge acquisition and self-perceived competence in response to immersive training. This design allows for the measurement of individual progress while minimizing inter-subject variability.

A further strength of our approach lies in the co-design of the VR scenarios with frontline healthcare professionals, ensuring that the training content is contextually relevant and aligned with real-world clinical communication challenges. This participatory development process enhances the ecological validity and acceptability of the training intervention.

Our approach also takes into account several critical facilitators and barriers to the effective implementation of VR-based training, as highlighted in recent literature. For instance, immersive VR has been shown to create a realistic and emotionally resonant learning environment that strengthens empathy and confidence among healthcare trainees [42,43]. However, limitations such as the lack of avatar interactivity or risk of cybersickness have been reported [43,44]. By relying on realistic scenarios with a human actor and structured debriefings, our design addresses some of these limitations while enhancing the sense of presence and pedagogical realism. Additionally, organizational aspects such as technical support and flexibility of use have been identified as key to successful implementation of VR programs in dementia care, and will be explored in future developments of this protocol [44].

In a systematic review based on the Cochrane methodology, Kyaw et al. explored medical literature on the effectiveness of VR for educating health professionals and improving their knowledge, cognitive skills, attitudes, and satisfaction in randomized and cluster randomized trials (31 studies between 1990 and 2017, 2407 participants) [53]. The authors found evidence suggesting that VR improves postintervention knowledge and skills outcomes of health professionals when compared with traditional education or other types of digital education such as online or offline digital education. However, they concluded that further research is needed to evaluate the effectiveness of immersive and interactive forms of VR and evaluate other outcomes such as satisfaction, cost-effectiveness, and clinical practice or behavior change.

Our study will allow us to meet some of these requirements. Additionally, through the co-construction of virtual scenarios, this project aims to facilitate the transfer of skill sets from healthcare professionals to industry and startup sectors.

While the study does not adopt a randomized controlled design, the pre-post methodology offers valuable insights into the training’s effectiveness, minimizing inter-individual variability and allowing each participant to serve as their own control. This approach is particularly suited for evaluating educational interventions under real-world clinical constraints.

## Limitations of the study design

There are a few limitations to our study design listed as following:

- Small sample size: The study involves only 40 healthcare professionals, which may limit the generalizability of the findings. Also, due to time constraints and availability, the sample size is not likely to increase.
- Single site study: The study is conducted at a single site, which may limit the generalizability of the results to other settings.
- Self-selection bias: The participants will be recruited voluntarily, which may introduce self-selection bias since those who are interested in the study may differ from those who are not interested.
- Lack of control group: The study design lacks a control group, which could be used to assess whether the observed effects are due to the training intervention or to other factors.
- Short duration of the study: The maximum duration of participation in the study is only 120 minutes, which may not be sufficient to assess the long-term effects of the training interventions.
- Limited outcome measures: The study will only evaluate the participants’ ability to identify appropriate and inappropriate verbal and nonverbal behaviors exhibited by healthcare professionals during interactions with patients who have BPSD. It does not assess whether the training interventions actually improve patient outcomes or quality of care.

### Dissemination plans

The dissemination plan for our study includes the following steps:

- Publishing the results in the peer-reviewed open access journal *PLOS One* if suitable to the editorial board. This will enable the research to reach a wider audience of healthcare professionals and researchers.
- Presenting at conferences and seminars: The study design and results of the study will be presented at conferences and seminars on geriatric nursing, communication skills, and dementia care. This will allow the research to reach a wider audience of healthcare professionals, researchers, and policymakers.
- Communicating with hospital management: The findings of this study will be shared with the management of Broca Geriatric Hospital in Paris to inform policy and practice. This could lead to changes in the training and education of healthcare professionals in the hospital, and potentially in other healthcare facilities and could integrate into the efforts made to improve the quality and safety of care provided to demented patients with disruptive behavioral and psychological symptoms.
- Creating infographics: The findings of the study can be transformed into infographics that are easy to read and share on professional media platforms and websites. This will enable the research to reach a wider audience of healthcare professionals, caregivers, and patients.
- Engaging with patient and caregiver groups: The findings of the study can be shared with patient and caregiver groups, such as the French or international Alzheimer’s Associations, to inform and improve dementia care. This will allow the research to have a direct impact on the lives of patients and their families.

### Management of study amendments and termination

Any amendments to this study protocol must be approved by the Institutional Review Board (IRB) before they can be implemented. The Principal Investigator (PI) will be responsible for submitting all proposed amendments to the IRB, along with a rationale and any necessary documentation. If amendments are approved, the PI will be responsible for ensuring that all study personnel are informed of the changes and that any necessary revisions are made to the study protocol, informed consent forms, and other study documents. If the study needs to be terminated for any reason, the PI will immediately inform the IRB, funders and all study personnel. The PI will also take appropriate measures to ensure the safety and well-being of all study participants and will work with the IRB to develop a plan for the collection and analysis of any existing data.

## Conclusion

In conclusion, our study protocol presents a new and innovative approach to training healthcare professionals in managing patients with disruptive behavioral and psychological symptoms of dementia. Through the utilization of virtual reality training, along with educational content provided in Moodle, trainees can learn from the mistakes of their virtual colleagues while interacting with a simulated patient. We believe that this approach will better prepare trainees to provide high-quality care to their patients. Additionally, the use of a real actor in the simulation further enhances the realism of the training experience, providing a more effective learning experience.

## Supporting information

S1 Checklist SPIRIT 2013 checklist. This file contains the SPIRIT 2013 checklist listing recommended items to address in a clinical trial protocol.
(PDF)

S2 Text Scenario: verbal and nonverbal interactions. This file presents two versions of a caregiver-patient interaction scenario, illustrating inappropriate and appropriate verbal and nonverbal communication behaviors.
(PDF)

S3 Text Communication training needs. This file provides a questionnaire assessing healthcare professionals’ training needs regarding communication with patients exhibiting behavioral and psychological symptoms of dementia.
(PDF)

S4 Text VR scenario satisfaction questionnaire. This file contains a satisfaction questionnaire evaluating participants’ perceptions of the virtual reality training scenario.
(PDF)

S5 Text Self-perceived competence. This file includes a questionnaire assessing healthcare professionals’ self-perceived competence in managing patients with behavioral and psychological symptoms.
(PDF)

S6 Text Trial oversight entities. This file describes the structure, composition, and responsibilities of the entities supervising the trial. vs: *versus.*
(PDF)

## Abbreviations

ANSES: Agence Nationale de Sécurité Sanitaire (French National Health Security Agency)
BPSD: behavioral and psychological symptoms of dementia
GDPR: General Data Protection Regulation
ID: Identification
IRB: Institutional Review Board
PI: principal investigator
V/NV: verbal and nonverbal
VR: Virtual Reality.
