# Supplementary material for: Feasibility, user satisfaction, and knowledge improvement after a VR training program for healthcare professionals managing behavioral and psychological symptoms of dementia (BPSD): Protocol for the FORMSPC-REALVI single-arm pre-post study
Source: PLoS One. 2025 Jun 10;20(6):e0325910. doi: 10.1371/journal.pone.0325910 (PMC12151340; doi:10.1371/journal.pone.0325910)
Supplement: S2 Text — This file presents two versions of a caregiver-patient interaction scenario, illustrating inappropriate and appropriate verbal and nonverbal communication behaviors. (PDF) [file pone.0325910.s002.pdf]

S2 File. Example of a scenario. Verbal and nonverbal interactions

First version. Poor and inappropriate verbal and non-verbal interactions

Mr. S is sitting on his armchair. His meal is served in front of him. Julien, the caregiver, enters Mr. S.'s room without knocking and notices that he has not touched his meal. He then says: "Good morning, Mr. S. Look at this, oh heaven, you haven't touched your meal yet? Well, let me feed you."
Julien stands next to Mr. S. With a sudden movement, he lifts the lid of his plate, starts mixing his food, and hastily feeds Mr. S. The latter keeps the pieces in his mouth. A nursing assistant arrives. Julien tells her, "I won't be able to attend the staff meeting because Mr. S. has decided to bother me by taking his time to eat." Julien gets frustrated with the time it takes Mr. S. to swallow. He tells him: "Come on, hurry up, we're not going to spend the whole afternoon here." Mr. S. then throws the meal tray to the ground and begins to hit Julien.

Second version. Suitable verbal and non-verbal interactions

Julien, the caregiver, enters Mr. S.'s room after knocking on the door and says: "Good morning, Mr. S." He sits next to Mr. S. on a chair, introduces himself by saying: "I am Julien, your nursing assistant," and gently extends his hand while smiling. He then says: "May I join you for lunch?" Mr. S. agrees by nodding his head. Julien then shows him each component of the meal, and says: "The meal looks good, you have soup, salad, meat and vegetables, and a dessert cream."
Julien then asks Mr. S.: "What would you like to eat first?" Since Mr. S. does not respond, Julien suggests starting with the soup. "Shall we start with the soup? Would you like to take your spoon?" Mr. S. no longer knows how to use the spoon. So, Julien gently takes Mr. S.'s hand and picks up the spoon, helping him drink his soup, then letting go of his hand and leaving the spoon in Mr. S.'s hand to encourage him to drink his soup without assistance. Julien encourages Mr. S. with every bite.
