# Supplementary material for: Feasibility, user satisfaction, and knowledge improvement after a VR training program for healthcare professionals managing behavioral and psychological symptoms of dementia (BPSD): Protocol for the FORMSPC-REALVI single-arm pre-post study
Source: PLoS One. 2025 Jun 10;20(6):e0325910. doi: 10.1371/journal.pone.0325910 (PMC12151340; doi:10.1371/journal.pone.0325910)
Supplement: S3 Text — This file provides a questionnaire assessing healthcare professionals’ training needs regarding communication with patients exhibiting behavioral and psychological symptoms of dementia. (PDF) [file pone.0325910.s003.pdf]

S3 File.

Verbal and nonverbal communication training needs' questionnaire

1. Profession

   Nurse ☐

   Certified nursing assistant ☐

2. Age : ---------------------- (years old)
3. Tenure in the profession : How long have you been working as a registered nurse or certified nursing assistant ?)

   For --------------------years

4. In the course of your professional practice, are you regularly in contact with patients with disruptive behavioral problems (agitation, aggressiveness, opposition, etc.)?

   Yes, occasionally ☐

   Yes, all the time or frequently ☐

   No ☐

5. In your professional training, have you ever dealt with the particularities of patients with Alzheimer's disease or related disorders?

   Yes ☐

   No ☐

6. Have you ever had difficulty communicating with a patient with Alzheimer's disease?

Yes ☐

No ☐

6.a. If so, in what type of situation? --

6.b. And what did you do to overcome these difficulties? ------------------------------------

----

7. In your professional training, have you been trained to verbal and nonverbal communication techniques with patients with Alzheimer's disease and/or related disorders?

Yes ☐

No ☐

8. If you have attended at least one of these training sessions, what teaching tools or methods were offered to you?

Theoretical course ☐

Pedagogical game (role play) ☐

Clinical case study and discussion ☐

Training via a digital tool (video games, internet training, virtual reality, etc.) ☐

Other ☐ , please specify --

9. Do you feel the need to improve your knowledge and skills by taking a training course on how to manage difficult situations with patients suffering from psychobehavioral disorders such as: agitation, aggression, opposition, etc.?

Yes ☐

No ☐

10. If yes, by what means would you like to acquire these skills or knowledges (rank from 1 to 4 according to your preference):

Theoretical course ☐

Clinical case study and discussion ☐

Educational game (role-playing) ☐

Training via a digital tool (video games, web-based training, virtual reality, etc.) ☐

Other ☐ , please specify --
