# Supplementary material for: Feasibility, user satisfaction, and knowledge improvement after a VR training program for healthcare professionals managing behavioral and psychological symptoms of dementia (BPSD): Protocol for the FORMSPC-REALVI single-arm pre-post study
Source: PLoS One. 2025 Jun 10;20(6):e0325910. doi: 10.1371/journal.pone.0325910 (PMC12151340; doi:10.1371/journal.pone.0325910)
Supplement: S4 Text — This file contains a satisfaction questionnaire evaluating participants’ perceptions of the virtual reality training scenario. (PDF) [file pone.0325910.s004.pdf]

S4 File.

VR scenario movie satisfaction questionnaire

Please read each statement and indicate on a scale from 1 (“Strongly disagree”) to 5 (“Strongly agree”) your level of agreement with the following statements:

1. The facial expressions and gestures of the virtual patient played by an actor seemed credible and natural.

Strongly disagree	1	2	3	4	5	Strongly agree
	◯	◯	◯	◯	◯	

2. I found that the behaviors and exchanges of the virtual healthcare professional with the virtual actor patient were credible, authentic, and natural.

Strongly desagree	1	2	3	4	5	Strongly agree
	◯	◯	◯	◯	◯	

3. I found that the content of the scenario corresponded to the clinical reality that I have observed in my professional life.

Strongly desagree	1	2	3	4	5	Strongly agree
	◯	◯	◯	◯	◯	

4. I felt uncomfortable or uneasy watching the interactions between the virtual healthcare professional and the virtual actor patient.

Strongly desagree	1	2	3	4	5	Strongly agree
	◯	◯	◯	◯	◯	

5. I felt comfortable not directly interacting with the virtual actor patient.

Strongly desagree	1	2	3	4	5	Strongly agree
	◯	◯	◯	◯	◯	

6. I was frustrated that I could not interact directly with the virtual actor patient.

Strongly desagree	1	2	3	4	5	Strongly agree
	◯	◯	◯	◯	◯	
