# Supplementary material for: Feasibility, user satisfaction, and knowledge improvement after a VR training program for healthcare professionals managing behavioral and psychological symptoms of dementia (BPSD): Protocol for the FORMSPC-REALVI single-arm pre-post study
Source: PLoS One. 2025 Jun 10;20(6):e0325910. doi: 10.1371/journal.pone.0325910 (PMC12151340; doi:10.1371/journal.pone.0325910)
Supplement: S5 Text — This file includes a questionnaire assessing healthcare professionals’ self-perceived competence in managing patients with behavioral and psychological symptoms. (PDF) [file pone.0325910.s005.pdf]

S5 File.

Self-perceived competence questionnaire

The notion of competence in healthcare can be defined as the set of knowledge, skills, and attitudes required to provide quality and effective care to a patient in a given healthcare environment. This includes the ability to assess the patient's needs, plan and implement appropriate interventions, communicate effectively with the patient and other members of the healthcare team, solve problems, make informed decisions, and reflect critically on one's own practice. Competence is therefore a crucial aspect in ensuring safe and quality care in all areas of healthcare.

Please read each statement and indicate on a scale from 1 (“Strongly disagree”) to 5 (“Strongly agree”) your level of agreement with the following statements:

1. Have you ever experienced “negative” emotions related to difficult communication when caring for a patient with Alzheimer's-type disorders? Such emotions may include frustration, anger, sadness, etc.

Strongly desagree	1	2	3	4	5	Strongly agree
	◯	◯	◯	◯	◯	

2. Has taking care of a patient with this type of pathology (dementia with disrupted behavioral and psychological symptoms) and communication disorders ever questioned your competence?

Strongly desagree	1	2	3	4	5	Strongly agree
	◯	◯	◯	◯	◯	

3. If so, why? And do you think you could have adjusted something?

----

4. After having participated in this training program, would you say that you have gained knowledge on how to manage your “negative” emotions in relation to communication difficulties with patients suffering from Alzheimer's-type disorders?

Strongly desagree	1	2	3	4	5	Strongly agree
	◯	◯	◯	◯	◯	

5. Following the training program, you have just participated in, do you feel more competent in taking care of patients with disruptive behavioral disorders such as aggressiveness, agitation and restless, care refusing, etc.?

Stronly desagree	1	2	3	4	5	Strongly agree
	◯	◯	◯	◯	◯	
