# Supplementary material for: Feasibility, user satisfaction, and knowledge improvement after a VR training program for healthcare professionals managing behavioral and psychological symptoms of dementia (BPSD): Protocol for the FORMSPC-REALVI single-arm pre-post study
Source: PLoS One. 2025 Jun 10;20(6):e0325910. doi: 10.1371/journal.pone.0325910 (PMC12151340; doi:10.1371/journal.pone.0325910)
Supplement: S6 Text — This file describes the structure, composition, and responsibilities of the entities supervising the trial. vs: versus. (PDF) [file pone.0325910.s006.pdf]

# S6 File- Composition, roles, and responsibilities of trial oversight entities

## Coordinating Centre (Project Coordinator)

**Composition**: One Project Coordinator, responsible for overseeing the entire study.

**Role**: Coordinate and manage the study, ensuring adherence to the protocol throughout all phases.

**Responsibilities**: Organize focus group sessions, facilitate collaboration among investigators, healthcare professionals, and participants, and ensure that the scenarios are pedagogically sound and aligned with the study objectives.

## Investigator Team (2 Psychologists and 2 Geriatricians)

**Composition**: A team composed of two psychologists and two geriatricians.

**Role**: Lead the scenario design phase, co-create scenarios with focus group participants, and contribute to the development of the Moodle-based theoretical reinforcement platform. •

**Responsibilities**: Facilitate focus group sessions, provide clinical expertise in scenario design, and collaborate in the creation of educational content for the Moodle platform.

## Virtual Reality Software Developers (Engineers from Broca Hospital's Research Lab)

**Composition:** Engineers specialized in virtual reality software development.

**Role:** Develop the virtual reality environment for the study.

**Responsibilities:** Translate the co-designed scenarios into a virtual reality format, ensure technical feasibility, and create a 3D immersive environment for the VR-based training modules.

## Healthcare Professionals (50 Participants)

**Composition**: Fifty healthcare professionals, including participants from the focus group and additional training participants.

**Role**: Actively participate in the focus group, propose modifications to scenarios, and participate in training sessions.

**Responsibilities**: Provide professional feedback during the focus group sessions, engage fully in VR-based training, complete evaluation questionnaires, and participate in pre- and post-training assessments.

## Psychologist Investigators

**Composition**: Psychologists involved in debriefing sessions.

**Role**: Conduct debriefing sessions, provide feedback to participants, and assist during training sessions.

**Responsibilities**: Discuss choices made during quizzes, offer constructive feedback, and facilitate post-assessment discussions and training sessions.

## Data Management Team

**Composition**: Team responsible for managing data collection and assessment tools.

**Role**: Oversee the administration of questionnaires and assessments, ensuring data integrity.

**Responsibilities**: Monitor data collection processes, organize and manage collected data, and support the assessment and analysis phases.

## Ethics Committee

**Composition**: External ethics committee.

**Role**: Review and approve the study protocol, ensuring ethical conduct throughout the study.

**Responsibilities**: Provide oversight on ethical considerations, participant safety, and adherence to applicable regulatory standards.

## Internal Monitor/Auditor

**Composition**: Independent internal monitor.

**Role**: Periodically review study conduct and internal data quality.

**Responsibilities**: Ensure protocol adherence and internal data integrity in compliance with Good Clinical Practice (GCP) guidelines.

This oversight structure ensures comprehensive management and coordination of the study, involving diverse expertise to guarantee the quality, ethical standards, and scientific validity of the research.
